# Non-coding RNAs Function as Immune Regulators in Teleost Fish

**DOI:** 10.3389/fimmu.2018.02801

**Published:** 2018-11-28

**Authors:** Man Wang, Shuai Jiang, Wei Wu, Fei Yu, Wenguang Chang, Peifeng Li, Kun Wang

**Affiliations:** ^1^Institute for Translational Medicine, Medical College of Qingdao University, Qingdao, China; ^2^Key Laboratory of Experimental Marine Biology, Institute of Oceanology, Chinese Academy of Sciences, Qingdao, China

**Keywords:** non-coding RNA, fish, infectious pathogen, immune response, immune regulator

## Abstract

Non-coding RNAs (ncRNAs) are functional RNA molecules that are transcribed from DNA but not translated into proteins. ncRNAs function as key regulators of gene expression and chromatin modification. Recently, the functional role of ncRNAs in teleost fish has been extensively studied. Teleost fish are a highly diverse group among the vertebrate lineage. Fish are also important in terms of aquatic ecosystem, food production and human life, being the source of animal proteins worldwide and models of biomedical research. However, teleost fish always suffer from the invasion of infectious pathogens including viruses and bacteria, which has resulted in a tremendous economic loss to the fishing industry worldwide. Emerging evidence suggests that ncRNAs, especially miRNAs and lncRNAs, may serve as important regulators in cytokine and chemokine signaling, antigen presentation, complement and coagulation cascades, and T cell response in teleost fish. In this review, we summarize current knowledge and understanding of the roles of both miRNAs and lncRNAs in immune regulation in teleost fish. Molecular mechanism insights into the function of ncRNAs in fish immune response may contribute to the development of potential biomarkers and therapeutic targets for the prevention and treatment of fish diseases.

## Introduction

Teleosts represent the largest and most diverse group of vertebrates, with an estimated number of species exceeding 25,000 (1). Teleosts are crucial in broad terms of ecology and food production. Teleosts also serve as biological models for developmental biology and genomic studies due to their high fecundity, rapid development, and ease of genetic manipulation (2, 3). However, teleost fish always suffer from the invasion of infectious pathogens including viruses and bacteria, which has caused a tremendous economic loss to the fishing industry worldwide. Therefore, it is urgent to identify effective biomarkers and therapeutic targets for the prevention and treatment of infectious fish diseases.

Non-coding RNAs (ncRNAs) are functional RNA molecules that are generally not translated into proteins (4). A growing body of evidence indicates that ncRNAs play a crucial role in diverse biological processes, such as development, differentiation and epigenetic regulation (5–7). In recent years, the research of teleost ncRNAs has been rapidly developed. Reportedly, teleost miRNAs are involved in multiple biological processes, including development, organogenesis, regeneration, tissue differentiation, growth and responses to environmental stimuli (8–11). LncRNAs have been found to be correlated with development and differentiation in teleost fish (12, 13). More importantly, the functional role of miRNAs and lncRNAs in fish immune responses has also been disclosed in several species (13–15). miRNAs and lncRNAs may serve as critical regulators in cytokine and chemokine signaling, antigen presentation, complement and coagulation cascades, and T cell response in teleost fish. In this review, we focus on the functional roles of miRNAs and lncRNAs in the regulation of fish immune responses. The advancement of our understanding about the immunoregulatory function of ncRNAs in teleost fish would facilitate the development of potential diagnostic markers and therapeutic targets for pathogenic diseases in economically important teleost fish species.

## The classification and function of ncRNAs

ncRNAs are mainly grouped into short/small ncRNAs and long ncRNAs (lncRNAs) based on their length (16). ncRNAs shorter than 200 nucleotides are usually defined as short/small ncRNAs, which include microRNAs (miRNAs), small nuclear RNAs (snRNAs), small nucleolar RNAs (snoRNAs), small interfering RNAs (siRNAs), transfer RNAs (tRNAs), and piwi-interacting RNAs (piRNAs) (17, 18). The most studied class of short ncRNAs is the miRNAs. miRNAs are single-stranded RNA molecules composed of approximately 18–24 nucleotides and can control gene expression by specifically targeting mRNAs (19). LncRNAs, which are usually longer than 200 nucleotides, are classified as long intronic ncRNAs, long intergenic ncRNAs, and natural antisense transcripts (20–23). Recently, a new group of ncRNAs, called circular RNAs (circRNAs), has been uncovered in various species (24–27). The length range and biological function of each type of ncRNAs are shown in Table 1. Their best characterized function is gene regulation. miRNAs can regulate gene expression by causing mRNA degradation or translational suppression (46). Like miRNAs, siRNAs, and piRNAs are able to mediate gene silencing by base pairing to the specific region of target mRNAs (47). LncRNAs are able to antagonize miRNA-mediated gene silencing by acting as competing endogenous RNAs (ceRNAs) (48).

**Table 1:** The category and function of non-coding RNAs.

**Non-coding RNA**	**Size**	**Functions**	**References**
miRNAs	18–24 nt	Post-transcriptional regulation	(28, 29)
snRNAs	100–300 nt	Pre-mRNA processing	(30, 31)
snoRNAs	60–300 nt	Nucleotide modification	(32, 33, 34)
siRNAs	20–30 nt	RNA interference	(35, 36)
tRNAs	74–95 nt	Transfer of amino acids during protein synthesis	(37, 38)
piRNAs	24–32 nt	Translational suppression, epigenetic regulation, transposon repression	(39, 40, 41)
lncRNAs	>200 nt	Gene expression regulation, epigenetic and chromatin structure modifications	(42, 43, 44)
CircRNAs	Circular	Gene expression regulation, miRNA sponge	(24, 45)

## The functional role of miRNAs in fish immune regulation

To date, a variety of miRNAs have been identified to be dysregulated in teleost fish during pathogen infection. Target genes of the aberrantly expressed miRNAs are prognosed through bioinformatics analysis. These dysregulated miRNAs might regulate the expression of genes that are involved in chemokine/cytokine signaling, TLR signaling, inflammatory response, complement and coagulation cascades, B- and T-cell mediated immune responses. Moreover, a few miRNAs have been further characterized by studying their effect on target genes (Table 2). Thus, increasing evidence confirms the important role of miRNAs in fish immune regulation. Further research is still needed to fully elucidate the regulatory function of miRNAs in fish immune response against pathogen infection.

**Table 2:** Overview of miRNAs characterized in teleost fish.

**miRNAs**	**Fish species**	**Targets**	**Function**	**References**
cse-miR-146a	Half-smooth tongue sole (*Cynoglossus semilaevis*)	IL-1β	Regulate inflammatory response	(49)
cse-miR-33	Half-smooth tongue sole (*Cynoglossus semilaevis*)	caspase-8	Regulate cell apoptosis	(49)
cse-let-7	Half-smooth tongue sole (*Cynoglossus semilaevis*)	IGFBP1	Regulate cell apoptosis	(49)
miR-148	Half-smooth tongue sole (*Cynoglossus semilaevis*)	SOCS7	Regulate cytokine signaling	(49)
cse-miR-143	Half-smooth tongue sole (*Cynoglossus semilaevis*)	ATG2B	Regulate cell autophagy	(49)
cse-miR-152	Half-smooth tongue sole (*Cynoglossus semilaevis*)	NLRC5	Regulate antigen processing	(49)
cse-miR-23a	Half-smooth tongue sole (*Cynoglossus semilaevis*)	STAT1	Regulate IFN signaling	(49)
cse-miR-26a	Half-smooth tongue sole (*Cynoglossus semilaevis*)	WIPI1	Regulate cell autophagy	(49)
cse-miR-71c-5p	Half-smooth tongue sole (*Cynoglossus semilaevis*)	IL-10	Regulate inflammatory response	(49)
cse-miR-8192-3p	Half-smooth tongue sole (*Cynoglossus semilaevis*)	WIPI1	Regulate cell autophagy	(49)
miR-148	Miiuy croaker (*Miichthys miiuy*)	MyD88	Regulate NF-κB signaling pathway	(50)
miR-214	Miiuy croaker (*Miichthys miiuy*)	MyD88	Regulate NF-κB signaling pathway	(51)
miR-3570	Miiuy croaker (*Miichthys miiuy*)	MyD88; MAVS	Regulate NF-κB and IRF3 signaling pathways	(52, 53)
miR-19a	Miiuy croaker (*Miichthys miiuy*)	MyD88	Regulate NF-κB signaling pathway	(54)
miR-192	Miiuy croaker (*Miichthys miiuy*)	IL-1RI	Regulate inflammatory response	(55)
miR-216a	Miiuy croaker (*Miichthys miiuy*)	p65	Regulate NF-κB signaling pathway	(15)
miR-203	Miiuy croaker (*Miichthys miiuy*)	IRAK4	Regulate NF-κB signaling pathway	(56)
miR-375	Miiuy croaker (*Miichthys miiuy*)	DUSP1	Regulate NF-κB signaling pathway	(57)
miR-146a	Fathead minnow (*Pimephales promelas*)	ND	Regulate cell apoptosis and NF-κB signaling pathway	(58)
miR-210	Miiuy croaker (*Miichthys miiuy*)	DUBA	Regulate RIG-I signaling pathway	(59)
miR-210	Miiuy croaker (*Miichthys miiuy*)	STING	Regulate type I IFN signaling pathway	(60)
pol-miR-731	Japanese flounder (*Paralichthys olivaceus*)	IRF7, p53	Regulate type I IFN response and cell apoptosis	(61)
miR-152	Antarctic ice-fish (*Chionodraco hamatus*)	GATA1	Regulate hematopoiesis	(62)
miR-146a	Orange spotted grouper (*Epinephelus coioides*)	TRAF6	Regulate inflammatory response	(63)
miR-126-03	Atlantic salmon (*Salmo salar*)	IFNg	Regulate IFN signaling pathway	(64)
miR-214	Snakehead fish (*Channa striatus*)	Viral N/P genes	Regulate SHVV propagation	(65)
miR-8159	Miiuy croaker (*Miichthys miiuy*)	TLR13	Mediate TLR signaling pathway	(66)
miR-8159-5p	Miiuy croaker (*Miichthys miiuy*)	TLR1	Mediate TLR signaling pathway	(67)
miR-217-5p	Miiuy croaker (*Miichthys miiuy*)	TLR1	Mediate TLR signaling pathway	(67)
miR-200a-3p	Miiuy croaker (*Miichthys miiuy*)	TLR1	Mediate TLR signaling pathway	(68)
miR-122	Miiuy croaker (*Miichthys miiuy*)	TLR14	Mediate TLR signaling pathway	(69)
miR-21	Miiuy croaker (*Miichthys miiuy*)	TLR28	Mediate TLR signaling pathway	(70)
cid-miRn-115	Grass carp (*Ctenopharyngodon idella*)	TLR5	Mediate TLR signaling pathway	(71)
miR-142a-3p	Grass carp (*Ctenopharyngodon idella*)	TLR5	Mediate TLR signaling pathway	(71)

*ND, not determined*.

### Differentially expressed miRNAs related to viral challenge

The miRNAs responsive to the viral mimic, polyriboinosinic polyribocytidylic acid (poly I:C), were identified in Atlantic cod through deep sequencing technology (14). The expression of ten miRNAs, including miR-731-3p, miR-462-3p, miR-2188-3p, miR-125b-3-3p, miR-150-3p, miR-128-3-5p, miR-214-1-5p, miR-451-3p, miR-30b-3p, and miR-199-1-3p, was confirmed to be altered upon poly I:C stimulation. Moreover, these differentially expressed miRNAs were predicted to target immune-related genes, such as interferon (IFN)-stimulated gene (ISG), CXC chemokine, and cytotoxic and regulatory T-cell protein precursor. Nevertheless, the number of sequencing reads mapped to miRNAs in the poly I:C stimulated samples was lower compared to the controls, which might affect the sensitivity of miRNA detection in Atlantic cod. Accordingly, several significantly deregulated miRNAs (e.g., miR-128-3-5p, miR-214-1-5p, and miR-731-3p) were not identified as differentially expressed by bioinformatics analysis. Remarkably, miR-462-3p, miR-731-3p, and miR-2188-3p have been reported as virus-responsive in several teleost species (72–74), suggesting that the three miRNAs were teleost-specific miRNAs upregulated by viral mimic stimulation. These miRNAs might exert similar functions in antiviral immune responses in teleost fish. The function of the identified miRNAs awaits further validation through loss- and gain-of-functional assays. Megalocytivirus, a highly infectious DNA virus, is a serious pathogen to a wide range of marine fish including Japanese flounder, catfish and rainbow trout (75–77). The expression profile of miRNAs was explored in Japanese flounder infected with megalocytivirus (78). The result indicated that 121 host miRNAs and 9 viral miRNAs were differentially expressed in virus-infected Japanese flounder. Functional categories showed that target genes of these miRNAs were predicted to be involved in immune response and apoptotic process. Specifically, apoptosis is an innate response of the host to counteract invading pathogens (79). The flounder miRNAs could target both pro- and anti-apoptotic genes, suggesting that some of the flounder miRNAs functioned in enhancing host defense responses, while others might operate to promote viral infection. It could be speculated that the differentially expressed host miRNAs might act on the host itself or virus. The host miRNAs might actively exert their functions in a natural manner that favored viral clearance. Oppositely, the host miRNAs could be subverted by the virus to promote viral infection. The effect of viral hemorrhagic septicemia virus (VHSV) infection on the miRNA expression profile in olive flounder was also studied (72). A total of 372 miRNAs were found, and 63 of these miRNAs were differentially expressed during VHSV infection. The predicted target genes of differentially expressed miRNAs were implicated in immune-related pathways such as cytokine-cytokine receptor interaction, nuclear factor κB (NF-κB) signaling pathway and tumor necrosis factor (TNF) signaling pathway. Notably, the most strikingly increased miRNAs during VHSV infection was miR-155. The putative target genes for miR-155 included the complement component C9 and the heat shock protein 90 (HSP90). HSP90 works as a molecular chaperone that promotes correct folding of target proteins (80). More importantly, HSP90 is also essential for viral replication (81, 82). The significant upregulation of HSP90-targeting miRNAs could be favorable or detrimental to olive flounder. Therefore, further studies should be conducted to disclose the role of the miRNA/HSP90 regulatory pathway in VHSV pathogenesis.

A total of 116 miRNAs were previously identified in *Epinephelus coioides* by the high-throughput sequencing approach (83). Among these miRNA, 107 miRNAs were identical in sequence to that of zebrafish. Moreover, 65 miRNAs had homologs in human, mouse, rat and pufferfish. The identified miRNAs in grouper were phylogenetically conserved. In the process of Singapore grouper iridovirus (SGIV) infection, 40 differentially expressed miRNAs were identified in *E. coioides*. During viral infection, host miRNAs underwent altered expression profiles, which led to feed-back or feed-forward effects on viral infection (84). The differentially expressed miRNAs were implicated in immune-related pathways including antigen processing and delivery, natural killer cell mediated cytotoxicity, and chemokine signaling pathway. These results might provide useful clues to uncover the mechanisms of host-virus interactions during SGIV infection. A total of 205 differentially expressed miRNAs were identified in snakehead fish vesiculovirus (SHVV)-infected snakehead fish cell line (85). Specifically, three differentially expressed miRNAs, including miR-130-5p, miR-214, and miR-216b, could inhibit the expression of viral genes. These miRNAs displayed significant antiviral properties. The three miRNAs might also target host genes that were involved in antiviral signaling pathways. It seemed that teleost miRNAs utilized both direct and indirect ways to inhibit SHVV multiplication. However, the detailed mechanisms underlying the inhibition of SHVV replication by teleost miRNAs remain to be further characterized. Another study revealed the miRNA response in rainbow trout following VHSV challenge (86). Two clustered miRNAs, miR-462, and miR-731, were strongly induced in rainbow trout following VHSV infection, indicating their engagement in the fish-virus interaction. In rainbow trout, the expression of miR-462 and miR-731 was positively correlated with the levels of IFNs and IFN-induced genes (87). Further study indicated that IFN stimulation could upregulate the expression of the two miRNAs, suggesting that the induction of miR-462 and miR-731 was elicited by IFNs. Knockdown of these miRNAs in rainbow trout treated with the IFN inducer poly I:C crippled the ability of poly I:C to protect fish against lethal VHSV infection. These findings implied that miR-462 and miR-731 might be involved in IFN-mediated antiviral defense in teleost fish. The true targets of these two miRNAs are currently undetermined and their regulatory effect on viral gene expression deserves further exploration. The miRNA response was identified in Atlantic salmon infected with salmonid alphavirus (SAV) (74). A total of 20 miRNAs showed altered expression during SAV infection. The differentially expressed miRNAs were predicted to target a variety of immune genes, such as IFN regulatory factors, C-C motif chemokines, cytokines, and NF-κB inhibitors. This study demonstrated that miRNAs functioned as inhibitors of harmful inflammation as well as promoters of early immune responses. The expression of these dysregulated miRNAs might differ depending on what kind of virus subtype that caused host immune responses. Additionally, the correctness of target gene prediction remains to be defined.

The expression pattern of miRNAs was characterized in epithelioma papulosum cyprini (EPC) cells following spring viremia of carp virus (SVCV) infection (88). A total of 14 miRNAs were found to be differentially expressed. Among their target genes, 51 genes were associated with IFN, interleukin, TNF and Toll-like receptor (TLR), all of which played important roles in host antiviral responses. Some differentially expressed miRNAs were predicted to target the coding region (CDS) of the SVCV genes, suggesting that these miRNAs might affect SVCV infection. Additionally, high-throughput sequencing combined with bioinformatics analysis was employed to characterize the function of immune-related miRNAs in miiuy croaker with poly I:C stimulation (89). Several upregulated miRNAs (e.g., mmi-miR-155, mmi-miR-181a-3p, mmi-miR-19a-3p, and mmi-miR-132-3p) identified in miiuy croaker had the potential to target inhibiting factors in the RIG-I like receptor (RLR) signaling pathway, thus, these miRNAs indirectly activated the signaling pathway. Moreover, miR-184, miR-200b-3p, and miR-30a-5p were predicted to negatively regulate the expression of caspase-8 (CASP8), c-Jun N-terminal kinase (JNK), IFN regulatory factor 3 (IRF3), and p38 mitogen-activated protein kinase (p38 MAPK). CASP8 participates in the regulation of apoptosis, while JNK, IRF3, and p38 MAPK can modulate cytokine production (90–92). miR-184, miR-200b-3p, and miR-30a-5p were downregulated in miiuy croaker, thus weakening their inhibitory effect on the target genes. Based on these results, miRNAs could indirectly activate fish immune responses through suppressing the inhibitory factor in signaling pathways. Moreover, attenuating miRNA-mediated silencing of immune-related genes resulted in the direct enhancement of fish immune responses. This study provided the opportunity to deeply understand the functional roles of miRNAs in fish immune regulation.

In these studies, a number of differentially expressed miRNAs have been identified in teleost fish. It is likely that most of differentially expressed miRNAs are not directly associated with fish immune regulation, but respond to external stimuli due to viral challenge. Some miRNAs may be even false positives. Nevertheless, if one miRNA is identified as differentially expressed in diverse teleost species infected with different viruses, this miRNA may serve as a vital modulator in fish immune system. For instance, miRNA-462/731 cluster and miR-2188 were conserved differentially expressed miRNAs in Japanese flounder and Atlantic cod following viral challenge, suggesting that these miRNAs played an important role in fish antiviral immunity. Notably, the miRNA-462/731 cluster is teleost-specific and locates downstream of IFN-inducible and immune-related promoter elements (86), indicating its crucial role in immune responses against viral infection in teleost fish. However, most of these studies focused on identifying miRNAs that were differentially expressed between the controls and challenged groups. The differentially expressed miRNAs were candidate miRNAs that might regulate immune-relevant genes. Experimental studies are demanded to validate the true targets of the deregulated teleost miRNAs during viral infection. Further investigation of teleost miRNAs as well as their target genes will deepen our understanding of the function of miRNA/mRNA regulatory axes in fish antiviral immunity.

### Fish virus-encoded miRNAs with immunomodulatory functions

Viruses have evolved their own miRNAs to regulate specific host pathways as a means for their survival and pathogenesis. The expression pattern of viral miRNAs was investigated in megalocytivirus-infected Japanese flounder (78). The result showed that nine viral miRNAs were upregulated and predicted to target immune-relevant genes including Toll like receptor 14 (TLR14), IRF3, and TNF receptor-1 (TNFR-1). None of the megalocytivirus miRNAs shared homology with known viral miRNAs. Viral miRNAs undergo fast evolution and mutation, which may render the virus capable of surviving in specific host environments. Yan et al. (93) found 16 novel viral miRNAs in SGIV-infected grouper cells through the high-throughput sequencing technology. These viral miRNAs were predicted to be engaged in viral proliferation and persistence, antiviral immune response and cell cycle regulation. This study might lay the foundation for further researches on SGIV pathogenesis.

Deep sequencing of the small RNAome in Cyprinus herpesvirus 3 (CyHV-3)-infected carps (*Cyprinus carpio*) revealed that CyHV-3 was able to encode two pre-miRNAs (94). Mature miRNAs from the two pre-miRNAs might target viral dUTPase. Since viral dUTPase can function as a signaling molecule to regulate antiviral immune responses (95), CyHV-3-encoded miRNAs might be involved in host immune regulation. CyHV-3 miRNAs might also target host mRNAs. The host target genes of CyHV-3 miRNAs could be identified once the *C. carpio* genome has been fully annotated. Among the SGIV miRNAs, SGIV-miR-13 exhibited enhanced expression during the early stage of SGIV infection (96). Overexpression of SGIV-miR-13 could significantly inhibit viral replication by targeting its major capsid protein (SGIV-MCP). Therefore, SGIV-miR-13 could effectively block host antiviral responses by restricting early viral replication. These results demonstrated that viruses have evolved their miRNA repertoire to escape host immune surveillance. At the late stage of viral infection, the expression of SGIV-miR-13 was decreased, thus relieving its inhibition of SGIV-MCP. Consequently, the downregulation of SGIV-miR-13 benefited viral replication. There was a tightly regulated kinetics of viral miRNA-mediated gene silencing during SGIV infection. Systematic explorations of SGIV-encoded miRNAs would provide novel insights into the molecular mechanism of SGIV pathogenesis. Host cells usually undergo apoptosis in response to viral infection, leading to reduced production and release of progeny virus (97). Viruses have evolved to manipulate host cell apoptosis. For instance, SGIV-encoded miR-homoHSV targeted viral pro-apoptotic lipopolysaccharide-induced TNF-α (LITAF)-like factor and suppressed LITAF-induced apoptosis (98). Therefore, miR-homoHSV inhibited SGIV-induced cell death and facilitated viral replication by targeting LITAF. These data demonstrated that viral miRNAs and protein-coding genes might work synergistically in a complicated manner to favor viral pathogenesis.

### Altered expression of miRNAs in response to bacterial challenge

Yuhong et al. (99) previously identified immune-related miRNAs in *Megalobrama amblycephala* using the high-throughput sequencing technology. As a result, 324 miRNAs were discovered, with 63 showed increased expression levels in lipopolysaccharide (LPS)-stimulated *M. amblycephala*. Functional annotation indicated that most of differentially expressed miRNAs were associated with immune system development and immune response, such as hematopoietic or lymphoid organ development, B or T cell-mediated immunity, TLR signaling pathway, complement and chemokine signaling pathway, and antigen processing and presentation. These results suggested that the differentially expressed miRNAs in *M. amblycephala* acted as important players in innate and adaptive immune responses against bacterial infection. Identification and functional characterization of immune-related miRNAs in *M. amblycephala* would be helpful for comprehensively disclosing the role of miRNAs in fish immune system. The miR-146 family, miR-146a, and miR-146b, was usually induced by *Salmonella typhimurium* or *Mycobacterium marinum* infection in zebrafish (100). Moreover, it was possible that the miR-146 family was involved in lipid-mediated inflammatory responses in zebrafish. Another study was conducted to investigate the miRNA expression profile in common carp infected with *Flavobacterium columnare* (FC) (101). Totally, 30 miRNAs were differentially expressed between the control and FC-infected common carp. These differentially expressed miRNAs were mainly enriched in pathways tightly associated with the bacterial infection, such as focal adhesion, erythroblastic leukemia viral oncogene homolog (ErbB) signaling pathway, adherent junction and regulation of actin cytoskeleton. These pathways were associated with cytoskeleton and its related signaling pathways, suggesting that FC infection exerted significant effects on cell proliferation, migration, adhesion, and differentiation in common carp. The exact mechanisms are unknown and remain to be elucidated. In addition, the differentially expressed miRNAs were also grouped into immune-related pathways including natural killer cell mediated cytotoxicity, TLR signaling pathway, transforming growth factor-β (TGF-β) signaling pathway, and mTOR signaling pathway. The immune mechanisms of these miRNAs involved in FC infection in common carp need further investigation.

The expression profile of the miRNAs response to *Vibrio anguillarum* infection was analyzed in *Cynoglossus semilaevis* (102). Compared to the controls, 175 and 215 miRNAs were differentially expressed in *C. semilaevis* with non-obvious and obvious vibriosis symptoms, respectively. Functional characterization indicated that the differentially expressed miRNAs might participate in immune system development and immune responses, such as hematopoietic or lymphoid organ development, T and B cell immune responses, TLR signaling pathway and chemokine-mediated signaling pathway. These miRNAs might play a central role in the modulation of immune-related genes in *C. semilaevis* during *V. anguillarum* infection. The expression pattern of miRNAs in Nile tilapia infected with *Streptococcus agalactiae* was also revealed (103). A total of 3,009 tilapia miRNAs were identified, and 218 miRNAs showed significantly altered expression in *S. agalactiae*-infected Nile tilapia. Target genes of tilapia miRNAs were prognosed to be implicated in T cell immune response, cytokine-mediated signaling pathway, antigen presentation, and the complement cascade. Some miRNAs (e.g., let-7, miR-21, miR-125, miR-146, and miR-155) were commonly deregulated during bacterial infection and functioned in reducing excessive inflammatory responses. let-7 and miR-125 exhibited high conservation across evolution in both sequence and function (104). Tilapia let-7 and miR-125 might possess the same function with other species, particularly in response to bacterial infection. Moreover, different tilapia miRNAs shared some target genes. In-depth investigation of synergistic regulations among diverse miRNAs will be crucial to uncover the mechanism of complex regulatory networks in tilapia during bacterial infection. Transcriptome sequencing analysis showed that 12 miRNAs were differentially expressed between *V. anguillarum*-infected miiuy croaker and the control group (105). Target genes of the 12 significantly deregulated miRNAs might engage in TLR signaling pathway, RLR signaling pathway, cytokine-mediated signaling pathway, and B or T cell immune responses. The relationship between miiuy croaker miRNAs and their target genes in host-pathogen interactions warrants further investigation. In addition, miR-142-5p, miR-223, and miR-181a were differentially expressed in *C. semilaevis* in response to *V. anguillarum* infection (106). Target genes of these miRNAs were related to B-cells, T-cells, NF-κB, TNF, cytokines and interleukin. Therefore, these miRNAs might serve as important players in fish immune responses against bacterial infection by regulating immune-related genes. Further studies on the function of miR-142-5p, miR-223, and miR-181a would enrich our knowledge about pathogen-induced defense responses in *C. semilaevis*.

The expression profile of miRNAs in *Edwardsiella tarda* infected-tongue sole was previously reported (49). The result indicated that 311 tongue sole miRNAs showed significant differences in expression after *E. tarda* infection. More importantly, target genes of 10 miRNAs were confirmed. For instance, cse-miR-146a could directly target *interleukin-1*β (*IL-1*β), while miR-148 regulated the expression of *suppressor of cytokine signaling 7* (*SOCS7*). Upregulated cse-miR-143 and cse-miR-152 targeted the autophagy-related gene *ATB2B* and the NOD-like receptor (NLR) gene *NLRC5*, contributing to *E. tarda* invasion into fish tissues. NLR and autophagy are implicated in recognition/clearance of invading pathogens (107, 108). Upregulation of cse-miR-143 and cse-miRNA-152 might be a virulence strategy of *E. tarda* to escape host immune defense by targeting the related pathways. Overall, miRNAs were not only vital immune regulators in tongue sole but might also act as targets for *E. tarda* manipulation of host defense system. A total of 1,894 miRNAs were discovered in *Streptococcus iniae-*infected tilapia (109). Among these miRNAs, seven miRNAs (miR-92d-3p, miR-127, miR-310-3p, miR-146-3p, miR-375-5p, miR-92, and miR-694) were found to be differentially expressed. Putative targets of these seven miRNAs included complement C3, cytidine deaminase, regulator of G-protein signaling 22 (Rgs22), MAPK1, calcium-sensing receptor (CaSR) and metabotropic glutamate receptor 8 (GluR8). These results suggested that tilapia miRNAs played an important role in host defense responses against bacterial infection. Further investigations are required to gain more insights into the relationship between miRNAs and their putative target genes in tilapia.

As mentioned above, teleost miRNAs may function as important regulators in fish immune responses against bacterial infection. Some teleost miRNAs, such as miR-462, miR-731, miR-181, and miR-146, were involved in the regulation of host immune responses against both bacterial and viral infection (73), suggesting that these miRNAs played a key role in host-pathogen interactions. However, target genes of multiple teleost miRNAs were predicted by computational approach. miRNA target predictions may be contaminated with a large number of false positives. The effect of predicted miRNA/mRNA regulatory axes on fish immune responses needs further functional experiments for validation.

### Target genes of teleost miRNAs involved in immune regulation

**Figure 1:**
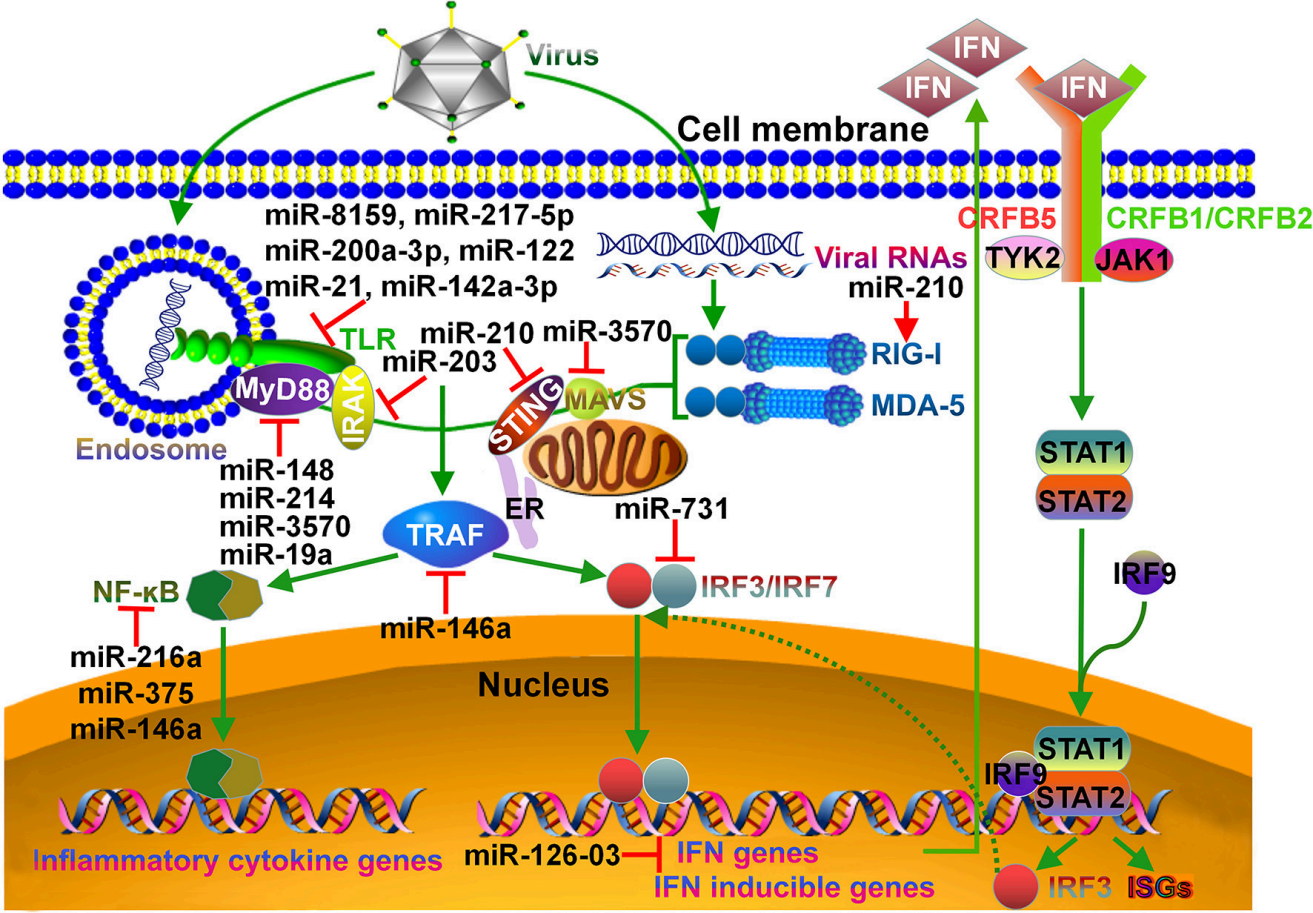
Schematic overview of miRNA targets in fish immune system. In teleost fish, viral infection can be detected by specialized pattern recognition receptors (PRRs). Endosome localized TLRs can sense viral nucleic acids (DNA, dsRNA, ssRNA), and then deliver signal to MyD88. MyD88 forms a complex with IRAK. MyD88/IRAK can activate the downstream TRAF signaling cascade, leading to the induction of NF-κB signaling pathway and pro-inflammatory cytokine response. RIG-I-like receptors, RIG-I and MDA-5, recognize viral genomic RNAs in the cytoplasm and signal via downstream STING, MAVS and TRAF adaptors. Both STING and MAVS can induce the phosphorylation of IRF3 and IRF7. Activated IRF3 and IRF7 translocate into the nucleus and bind to DNA sequences to induce the transcription of IFNs and IFN inducible genes. The induced IFN can bind to the CRFB complex on the cell surface, resulting in the recruitment and binding of the kinases TYK2 and JAK1. These kinases catalyze the phosphorylation of STAT1 and STAT2. The IRF9/STAT1/STAT2 complex crosses the nuclear membrane and binds to IFN-stimulated response elements, thereby contributing to the transcription of IRF3 and ISGs. miRNAs that regulate particular immune pathways are indicated. TLR, Toll-like receptor; MyD88, myeloid differentiation primary response 88; IRAK, interleukin-1R-associated kinase; STING, stimulator of IFN genes; ER, endoplasmic reticulum; MAVS, mitochondrial antiviral signaling protein; RIG-I, retinoid acid inducible gene-I; MDA-5, melanoma differentiation-associated gene-5; TRAF, tumor necrosis factor receptor-associated factor; IRF, IFN regulatory factor; CRFB, cytokine receptor family B; STAT, signal transducer and activator of transcription; ISG, IFN-stimulated gene.

Target genes of substantial teleost miRNAs have been confirmed and are shown to be widely involved in fish immune responses, including NF-κB signaling pathway, TLR signaling pathway and cytokine-mediated immune response (Figure 1). NF-κB plays a central role in activation of host pro-inflammatory response (110). Several teleost miRNAs were found to interfere with NF-κB-mediated inflammatory response. The expression of miR-148 (50) and miR-214 (51) was increased in miiuy croaker after challenged with *Vibro harveyi* or LPS, and their upregulation could restrain host inflammatory responses. Moreover, miR-148 and miR-214 were shown to negatively regulate the expression of myeloid differentiation primary response 88 (MyD88). miR-148 and miR-214 suppressed NF-κB signaling pathway by directly targeting MyD88, thus avoiding excessive inflammation. Likewise, miR-3570 suppressed *V. anguillarum*-induced inflammatory response in miiuy croaker by inhibiting MyD88-mediated NF-κB signaling pathway (52). Another report showed that the expression of miR-3570 was induced in miiuy croaker macrophages upon rhabdovirus infection (53). miR-3570 directly targeted mitochondrial antiviral signaling protein (MAVS), thus leading to the blockage of MAVS-mediated NF-κB and IRF3 signaling pathway. Further study indicated that inducible miR-3570 also suppressed virus-triggered type I IFN and antiviral gene production, hence favoring rhabdovirus pathogenesis. Recently, miR-19a was also reported to restrain NF-κB activation by targeting MyD88 in miiuy croaker (54). In teleost fish, multiple miRNAs may synergistically target NF-κB signaling pathway, hence significantly repressing host inflammatory responses.

miR-192 exhibited differential expression patterns between *V. anguillarum* challenge and healthy miiuy croakers (55). More importantly, miR-192 was confirmed to regulate inflammatory immune responses in miiuy croaker with bacterial infection by targeting IL-1 receptor type I (IL-1RI). Another two miRNAs, miR-216a (15), and miR-203 (56), were obviously upregulated in miiuy croaker challenged with *V. anguillarum* and LPS. miR-216a caused p65 downregulation by binding to its 3′ untranslated region (3′ UTR). Further study indicated that upregulation of miR-216a inhibited inflammatory cytokine production and blocked NF-κB signaling pathway. Thus, miR-216a functioned as a critical inhibitor of bacterial infection-induced inflammatory responses in miiuy croaker. IL-1 receptor-associated kinase 4 (IRAK4) was the target of miR-203. miR-203 repressed the activation of NF-κB signaling pathway by reducing the expression of IRAK4. These results suggested that miR-203 in miiuy croaker was a negative modulator of innate immune response against bacterial infection. Additionally, miR-375 significantly inhibited the expression of dual-specificity phosphatase 1 (DUSP1) in miiuy croaker and thus resulted in the blockade of NF-κB signaling pathway (57). Another study revealed the regulatory function of miR-146a in fathead minnow cells infected with SGIV (58). SGIV infection boosted the expression of miR-146a. miR-146a overexpression suppressed NF-κB activation and SGIV-induced cell apoptosis, thus promoting viral proliferation. These results indicated that miR-146a served a promotive role in SGIV infection by inhibiting host antiviral immune responses.

It has been reported that the expression of miR-210 was significantly upregulated in miiuy croaker upon poly I:C stimulation (59). The upregulation of miR-210 could indirectly induce the RIG-I signaling pathway and enhanced the expression of type I IFN by targeting deubiquitinating enzyme A (DUBA), thus protecting the host from viral infection. Likewise, miR-210 was found to be upregulated in *Siniperca chuatsi* rhabdovirus (SCRV)-infected miiuy croaker (60). However, further study indicated that miR-210 inhibited type I IFN and inflammatory cytokine production by targeting stimulator of IFN genes (STING) and repressing NF-κB, IRF3 and IFN-stimulated response element (ISRE) signaling. The regulatory function of miR-210 in STING expression was also verified in other teleosts (*Larimichthys crocea* and *Sciaenops ocellatus*). This result demonstrated the evolutionary conservation and functional consistency of miR-210 in teleosts. Type I IFNs are early host responses to viral infection and exert important roles in antiviral process. Thus, miR-210 functioned in supporting viral infection in teleost fish. It was possible that the host miR-210 could be subverted by SCRV to facilitate its infection. In summary, miR-210 could target diverse genes, and its target genes might have opposite effects on viral pathogenesis. The effect of miR-210 on type I IFN signaling might differ depending on the type of stimulus (poly I:C or SCRV), which further indicated the complexity of miRNA-mediated regulatory networks in teleost fish. The flounder miRNA, pol-miR-731, was upregulated upon megalocytivirus challenge and promoted viral replication (61). Further study showed that pol-miR-731 blocked type I IFN response by targeting IRF7, and it also suppressed virus infection-induced splenocyte apoptosis and cell cycle arrest by targeting p53. This study revealed the immuno-suppressive role of miRNAs in the host-virus interaction. The replenishment of hemocytes by hematopoiesis was of great significance in maintaining immune homeostasis (111). miR-152 performed a crucial role in hematopoiesis during the development of *Chionodraco hamatus* (62). Mechanistically, miR-152 caused decreased hematopoiesis by descending the expression of GATA-binding factor 1 (GATA1). Red spotted grouper nervous necrosis virus (RGNNV) infection constantly induced the expression of miR-146a in spleen cells of orange spotted grouper, *Epinephelus coioides* (63). miR-146a overexpression could promote RGNNV infection, while knockdown of miR-146a expression inhibited viral infection. Moreover, miR-146a specifically bound to the 3′ UTR of TNF receptor-associated factor 6 (TRAF6) and thereby attenuated the expression of downstream genes, including TNF-α, IL-8, and IL-1β. Collectively, these results showed that miR-146a exerted an inhibitory function in TRAF6-mediated inflammatory response to viral infection in orange spotted grouper. The previous study indicated that IFNg acted as a putative target of miR-126-03 in Atlantic salmon (64). Further study verified that miR-126-03 directly regulated the expression of IFNg. miR-214 was able to inhibit SHVV replication in striped snakehead cells by directly targeting viral nucleoprotein (N) and phosphoprotein (P) genes (65). SHVV infection led to decreased expression of miR-214. The downregulated miR-214 in turn promoted SHVV replication by enhancing viral N and P expression and reducing IFN-α production. This study provided new insights into the molecular mechanisms underlying fish immune responses against viral infection.

Toll-like receptors (TLRs), a group of pattern-recognition receptors, play an important role in modulating innate immune responses against invading pathogens (112). TLR13 is significantly enhanced in miiuy croaker following infection with *V. anguillarum* (66). miR-8159 was induced in miiuy croaker challenged with *V. anguillarum* and exerted a direct negative regulatory effect on TLR13. These results demonstrated that miR-8159 inhibited fish immune responses by targeting TLR13, thus facilitating bacterial infection and pathogenesis. TLR1 performs a crucial role in sensing bacterial pathogens and anti-microbial immune responses (113). miR-8159-5p and miR-217-5p were found to be upregulated in miiuy croaker upon LPS stimulation (67). The two miRNAs synergistically suppressed the expression of TLR1 response to LPS stimulation. miR-200a-3p could also directly target TLR1 in miiuy croaker and thus regulated fish TLR signaling pathways (68). TLR14 has only been discovered in fish and may be involved in fish immune responses against pathogen infection (114). miR-122 displayed significantly reduced expression profiles in *V. anguillarum* challenged miiuy croaker (69). miR-122 was involved in TLR cascade by targeting TLR14 and thus controlled *V. anguillarum* infection in miiuy croaker. TLR28 is a new member of TLRs family and plays an important role in host immune responses (115). The previous report showed that the expression of miR-21 was elevated in miiuy croaker after poly I:C stimulation (70). Upregulation of miR-21 inhibited the expression of cytokines (TNFα and IL-6) and antiviral genes (*MX1* and *ISG15*). miR-21 also suppressed the excessive immune response and maintained body homeostasis in miiuy croaker by directly targeting TLR28. The activation of TLR5 could modulate the expression of genes involved in innate immune responses (71). cid-miRn-115 and miR-142a-3p were differentially expressed between susceptible and resistant grass carps infected with *Aeromonas hydrophila*. Upregulation of cid-miRn-115 and miR-142a-3p significantly reduced the expression of TLR5, thus suppressing its downstream effector genes including IL-8, TNF-α, and IL-1β. These results demonstrated that cid-miRn-115 and miR-142a-3p played a crucial role in governing the innate immune response.

In the process of pathogen infection, the host immune responses are triggered to resist pathogen infection. However, to establish a persistent infection, pathogens have evolved their capability to escape host immune responses. In teleost fish, miRNAs serve as crucial effectors in the modulation of host-pathogen interaction networks. Intriguingly, the implication of miRNAs not only could be a part of host defense responses against pathogen infection, but host miRNAs could also be exploited by pathogens to support their replication. A better understanding of the functional roles of host miRNAs in immune regulation during pathogen invasion would shed light on the development of novel therapies for fish diseases.

### The engagement of LncRNAs in fish immune response

LncRNAs have been demonstrated to function as important regulators of gene expression (116). They are able to cis-regulate the expression of their neighboring protein-coding genes (117). LncRNAs may be involved in biological processes similar to those regulated by their neighboring protein-coding genes (118). Thus, the biological function of the uncharacterized lncRNAs can be predicted based on the functionality of the nearby protein-coding genes. LncRNAs have been confirmed to participate in multiple processes, such as cell proliferation, development and diseases (119). LncRNAs are also involved in a variety of immune responses including inflammatory response and IFN-mediated response (120–122). However, most of the studies in the lncRNA field are limited to mammalian species. Currently, researchers are showing more interest in inquiring into the role of lncRNAs in teleost fish. Due to the development of high-throughput sequencing technology, the functional role of lncRNAs in fish immune response against pathogen infection has been gradually disclosed.

Previously, Boltaña et al. (123) reported the transcriptomic regulation of lncRNAs in Atlantic salmon during infectious salmon anemia virus (ISAV) infection. A total of 5,636 putative lncRNAs were identified, most of which were regulated in response to ISAV infection. Intriguingly, most of the lncRNAs were similarly regulated in response to ISAV infection and were enriched in genes correlated with innate immunity and antigen presentation. Remarkably, seven lncRNAs (Ss_lncRNA_575, Ss_lncRNA_1421, Ss_lncRNA_1969, Ss_lncRNA_2198, Ss_lncRNA_2753, Ss_lnRNA_4968 and Ss_lncRNA_4977) were strongly associated with antiviral mRNA expression. The expression of Ss_lncRNA_2198 was highly correlated with grass carp reovirus (GCRV)-induced gene 2 (*GIG2*) (124). Taken together, these results demonstrated that lncRNAs might be implicated in host defense response to ISAV infection. This study would be helpful for fully uncovering lncRNA regulation during ISAV pathogenesis. LncRNAs also affect the activity of miRNAs. The deregulated lncRNAs, miRNAs, and their target genes might constitute complex modulatory networks in the defense response to ISAV infection. Mechanistic characterization of the lncRNA/miRNA/mRNA axes will be beneficial to expand our understanding of viral pathogenesis in teleost fish. C. Gallardo-Escárate' research group previously revealed the ncRNA response during *Piscirickettsia salmonis* infection in Atlantic salmon (125). A total of 918 putative lncRNAs were identified, 425 of which were newly characterized for Atlantic salmon. These lncRNAs were strongly correlated with clathrin-mediated endocytosis and iron homeostasis, demonstrating a potential mechanism of immune evasion by *P. salmonis*. This research group further profiled a widespread differential expression of lncRNAs in Atlantic salmon infected with different pathogens including ISAV, *P. salmonis* and the ectoparasite copepod *Caligus rogercresseyi* by comparative transcriptome analysis (126). The result showed that lncRNAs were widely regulated during pathogen infection, and this regulation was pathogen-specific and tightly associated with immune-related genes involved in innate immunity. LncRNAs have a broader spectrum of regulation, and serve as versatile regulators of gene expression (127). Intriguingly, a single lncRNA can mediate both the activation and suppression of immune responses (120). Both positive and negative transcriptional association between lncRNAs and their target genes could be expected in teleost fish. Further functional analyses are required to fully characterize the regulation of lncRNAs in the transcriptional responses of Atlantic salmon to invading pathogens.

A comprehensive study was previously performed to identify putative lncRNAs in the rainbow trout genome (128). A total of 54,503 putative lncRNAs were discovered. Of the putative lncRNAs, 2,935 lncRNAs displayed tissue-specific expression pattern. Moreover, the functional prediction indicated that several lncRNAs were positively associated with NF-κB inhibitor-like protein and MAPK1. However, the detailed mechanism underlying the regulation of genes by lncRNAs requires to be further surveyed. Moreover, a total of 9,674 large intergenic ncRNAs (lincRNAs) were previously found in rainbow trout based on the analysis of RNA-sequencing data (13). The predicted rainbow trout lincRNAs shared similar genomic features with lincRNAs from other species, such as human, mouse, and zebrafish. The lincRNAs exhibited tissue-specific expression pattern and were typically co-expressed with their adjoining genes. By clustering associated genes together, 34 co-expression gene modules consisting of 2,963 lincRNAs and 10,321 protein-coding genes were identified. Notably, 6 of 34 modules were associated with immune responses. LincRNAs in the 6 modules were co-expressed with genes implicated in T cell receptor signaling, immune cell activation, and antigen processing and presentation. The expression profile of lncRNAs in the intestine of rainbow trout was also analyzed, and hundreds of lncRNAs were differentially expressed in trout fed with functional diets based on pre- and probiotics compared to the control group (129). Some differentially expressed lncRNAs might regulate the expression of immune-related genes including TLR and TNF-α.

The expression profile of lncRNA was previously determined between rainbow trout strains, one a control strain and the other a strain fed an all plant-protein diet (130). A total of 142 unique antisense lncRNAs were found to be differentially expressed between the strains, 60 of which overlapped with genes involved in lipid metabolism and immune functionality, such as sterol regulatory element binding protein 1, sterol carrier protein 2, MHC class I heavy chain precursor, complement components C3 and C9, and IFN inducible protein 2. The differentially expressed lncRNAs in response to *Flavobacterium psychrophilum* challenge were analyzed in three genetic lines of rainbow trout (131). As a result, 556 differentially expressed lncRNAs were identified. Some differentially expressed lncRNAs were found to be strongly correlated with immune-relevant genes including complement components, and cytokines and chemokines. Several differentially expressed lncRNAs were associated with signaling molecules implicated in microbial infection processes, mTOR signaling pathway, T cell immune response and others. This study indicated that lncRNAs were able to control anti-bacterial immune responses in rainbow trout. A catalog of lncRNAs in the large yellow croaker has been discovered by characterization of the transcriptome in spleen, muscle, liver, and egg tissues (132). Accordingly, a total of 210 lincRNAs were identified in *V. anguillarum*-infected spleen tissue of the large yellow croaker. These lincRNAs were predicted to be engaged in immune responses, such as positive regulation of leukocyte/lymphocyte and T-cell activation. Specifically, two lincRNAs, Linc_08129.1 and Linc_32654.1, had the potential to activate the TLR signaling pathway to elicit host antiviral responses. The expression of four lincRNAs, Linc_00388.1, Linc_01738.1, Linc_06498.1, and Linc_09316.1, was elevated at 24 h after *V. anguillarum* infection and was then decreased at 48 h. This result implied that the large yellow croaker lincRNAs tended to participate in the early stage of host immune responses. The depth of RNA sequencing used in this study might hamper the discovery of lncRNAs with low abundance. Further identification of lncRNAs in the large yellow croaker is required.

Low sequence conservation of lncRNAs has limited the elucidation of lncRNA function based on comparative sequence analysis. Further study on the structural and regulatory elements of lncRNAs would aid the identification and characterization of lncRNAs in teleost fish. Currently, the identification of lncRNAs largely depends on the bioinformatics analysis. The computational tools might mis-identify the lncRNAs harboring short conserved open reading frame (ORF) regions as protein-coding transcripts. Conversely, protein-coding transcripts containing short conserved ORFs might be mis-identified as lncRNAs. Ribosome profiling can be employed to assess the protein-coding characteristics of putative lncRNAs (133). High-throughput RNA sequencing and ribosome profiling could be applied to identify genuine lncRNAs in teleost fish.

LncRNAs have been shown to play central roles in mRNA processing, gene expression and epigenetic regulation (134). LncRNAs may represent a new layer of gene expression modulation responsible for controlling host immune responses against pathogen infection (135). More importantly, lncRNAs possess the potential to serve as a new class of diagnostic biomarkers for fish diseases. The recent discovery of immune-related lncRNAs in teleost fish has opened up a new era of immune regulation exploration. Characterization of lncRNAs correlated with host defense responses would have deep impacts in the field of fish immunology. Functional crosstalk between lncRNAs and miRNAs in fish immune regulation is an important area of interest. Determining the roles of immune-related lncRNAs in teleost fish will facilitate our comprehension of their functional roles in immune responses. In-depth investigations on lncRNA regulation and function are warranted to comprehensively reveal the mechanism underlying modulation of fish immune responses by lncRNAs.

### Identification of circRNAs in teleost fish

Circular RNAs (circRNAs), a novel type of ncRNAs, are characterized by covalently closed loop structures with neither 5′ end caps nor 3′ poly (A) tails (136). CircRNAs are evolutionarily conserved across multiple species, and display tissue-specific and development-dependent expression patterns (137). The function of circRNAs has become a research hotspot in recent years. Emerging evidence indicates that circRNAs are master regulators of gene expression by acting as miRNA sponges and perform important roles in a variety of biological processes (138–140). CircRNAs also fulfill regulatory function in host immune responses (141, 142). Increasing transcriptome and phenotype data enable us to explore the biological function of circRNAs in teleost fish.

The identification of circRNAs from RNA-sequencing data in the large yellow croaker has been reported (143). A total of 975 circRNAs were detected, and the circRNA-host genes were enriched in translational progression, binding-related functions, RNA degradation and transport, and metabolic pathways. Intriguingly, KEGG enrichment showed that the circRNA-host genes were significantly enriched in complement and coagulation cascades, and antigen processing and presentation. The circRNA-host genes had at least two different circRNA isoforms, demonstrating that circRNAs had alternative circulations in the large yellow croaker. The regulation and formation mechanisms for alternative circularization of circRNAs in the large yellow croaker may be complex and need systematic study in the future. In addition, a number of circRNAs were predicted to harbor more than one miRNA binding sites. These circRNAs might have a broad impact in immune regulation by sponging miRNAs in the large yellow croaker. This study provided a valuable reference for circRNA study in teleost fish. Deep illumina sequencing was performed to discover the circRNAs in GCRV-infected grass carp (144). As a result, 5,052 novel circRNAs were identified in grass carp in response to viral infection. Moreover, 41 of the identified circRNAs were differentially expressed. Further analysis showed that 72 miRNAs were predicted to interact with the differentially expressed circRNAs. Target genes of the binding miRNAs mainly took part in immune response, hemostasis, blood coagulation, and complement and coagulation cascades. These results demonstrated that the circRNA-miRNA-mRNA axes existed in virally infected grass carp, providing novel insights into the molecular mechanism underlying pathogen infection and fish immune regulation. CircRNAs in zebrafish were sequenced and identified using the high-throughput sequencing technology combined with bioinformatics approach (145). A total of 3,868 circRNAs were found in zebrafish, 1,122 of which exhibited homology with human, mouse and coelacanth circRNAs. Accordingly, study on teleost circRNAs would also accelerate our efforts to fully elucidate circRNA-mediated regulatory networks in human owing to high conservation of circRNAs.

These studies provided valuable genetic resources for basic research and aquaculture improvement of teleost fish. The functional roles of circRNAs are receiving increasing attention. CircRNAs played an important role in antiviral immunity (146). It is likely that circRNAs function as pivotal regulators in fish defense system. However, few studies on circRNAs have been reported in teleost fish. More studies are needed to mine and characterize circRNAs in fish species. The detailed exploration of circRNA functions would shape our understanding of circRNA biology in teleost fish.

## Concluding remarks

In recent years, great progress has been made in elucidating the function of ncRNAs in fish immune responses which would benefit the rapid expanding aquaculture industry worldwide. Identification of immune-related ncRNAs in teleost fish is mainly based on the high-throughput sequencing technology. High-throughput sequencing is a powerful platform for the genome-wide identification of novel ncRNAs. The number of sequencing reads that are mapped to a particular ncRNA directly reflects the abundance of this ncRNA, indicating that high-throughput sequencing data can indicate the relative expression of identified ncRNAs. Thus, high-throughput sequencing results can be used for quantitative analysis of ncRNAs. Based on these clues, high-throughput sequencing platform seems to be the best choice for comprehensive profiling of ncRNAs in teleost fish. Besides, online resources and bioinformatics analysis are essential for the study on ncRNA biology and function.

A number of miRNAs have been found to control host immune responses by targeting immune-relevant genes in teleost fish. The explicit roles of miRNA/mRNA regulatory axes in fish defense responses need further exploration. In addition, lncRNAs and circRNAs have been confirmed to offset miRNA-mediated gene silencing by acting as ceRNAs. It will be intriguing to investigate the functional roles of lncRNA/miRNA/mRNA or circRNA/miRNA/mRNA regulatory networks in immune regulation. Further study on the biology of teleost ncRNAs will contribute to our understanding of the complex interaction between the regulatory ncRNAs and fish immune system during the process of pathogen infection.

Nevertheless, there is a lack of high quality reference genome/transcriptome resources for teleost fish, which restricts the ncRNA research in teleost fish. Meanwhile, the miRNAomes of fish species are incomplete. The identification and functional characterization of ncRNAs are crucial to increase our systematical understanding of the biological processes underlying teleost development, physiology and immunity. Moreover, investigations on teleost genetic regulatory elements, including miRNAs, lncRNAs, and circRNAs, would provide new insights into human gene regulatory networks through orthologous gene functional studies. Furthermore, increasing evidence suggests that ncRNAs hold great promise to be used as novel biomarkers for the control and treatment of fish diseases. Therefore, more research has to be focused on teleost ncRNAs which would accelerate the development of diagnostic markers and therapeutic targets for pathogenic diseases in economically important teleost fish species.

So far, a variety of ncRNAs have been identified in teleost fish through the high-throughput sequencing technology. Target genes of these teleost ncRNAs are predicted through bioinformatics analysis. Therefore, functional validation using experimental approaches will be the next important step in the research on ncRNAs associated with immune responses in teleost fish. With the growing number of available sequenced genomes, the functional roles of ncRNAs in fish immune responses will be better understood in the future.

## Author contributions

MW wrote the manuscript. SJ, WW, and FY prepared tables and figures. WC, PL, and KW edited the manuscript.

### Conflict of interest statement

The authors declare that the research was conducted in the absence of any commercial or financial relationships that could be construed as a potential conflict of interest.
